# The effects of differential feeding on ileum development, digestive ability and health status of newborn calves

**DOI:** 10.3389/fvets.2023.1255122

**Published:** 2023-09-08

**Authors:** Jie Wang, Yang Chen, Mianying Li, Siqi Xia, Kaisen Zhao, Huimei Fan, Jiale Ni, Wenqiang Sun, Xianbo Jia, Songjia Lai

**Affiliations:** College of Animal Science and Technology, Sichuan Agricultural University, Chengdu, China

**Keywords:** calves, feeding patterns, metagenomics, metabolomics, neuro microbiology

## Abstract

Pre-weaning is the most important period for the growth and development of calves. Intestinal morphology, microbial community and immunity are initially constructed at this stage, and even have a lifelong impact on calves. Early feeding patterns have a significant impact on gastrointestinal development and microbial communities. This study mainly analyzed the effects of three feeding methods on the gastrointestinal development of calves, and provided a theoretical basis for further improving the feeding mode of calves. it is very important to develop a suitable feeding mode. In this study, we selected nine newborn healthy Holstein bull calves were randomly selected and divided into three groups (*n* = 3), which were fed with starter + hay + milk (SH group), starter + milk (SF group), total mixed ration + milk (TMR group). After 80 days of feeding Feeding to 80 days of age after, the ileum contents and blood samples were collected, and the differences were compared and analyzed by metagenomic analysis and serum metabolomics analysis. Results show that compared with the other two groups, the intestinal epithelium of the SH group was more complete and the goblet cells developed better. The feeding method of SH group was more conducive to the development of calves, with higher daily gain and no pathological inflammatory reaction. The intestinal microbial community was more conducive to digestion and absorption, and the immunity was stronger. These findings are helpful for us to explore better calf feeding patterns. In the next step, we will set up more biological replicates to study the deep-seated reasons for the differences in the development of pre-weaning calves. At the same time, the new discoveries of neuro microbiology broaden our horizons and are the focus of our future attention.

## 1. Introduction

Pre-weaning is the most important period for the growth and development of calves. Intestinal morphology, microbial community and immunity are initially constructed at this stage, and even have a lifelong impact on calves (1, 2). The living environment of the calf changed from a sterile uterus to the outside world, and the nutritional conditions changed from maternal provision to the calf’s own feeding of feed and milk. Due to the incomplete development of the immune ability and digestive system of calves, newborn calves are very sensitive to external stimuli, and any external interference will seriously affect the growth of calves (3, 4). If the appropriate feeding mode is not adopted at this time, it will lead to low daily weight gain, diarrhea (5). It is reported that 30% of the deaths of pre-weaning cows are caused by diarrhea, which highlights the importance of intestinal health (6). The development of the digestive tract of calves is a unique process. With the development of the gastrointestinal tract and the settlement of the microbial community, calves gradually change from pseudo-ruminant animals to functional ruminants in physiology. The morphology of intestinal epithelial villi, the development of intestinal smooth muscle and rumen volume are basically completed at this stage which was ac-companied by the development of salivary organs and the development of rumination behavior. These factors directly affect the feed intake, nutrient absorption rate and digestive ability of calves after weaning (7).

Studies have shown that early feeding patterns have a significant impact on gastrointestinal development and microbial communities. Differences in dietary composition and feed physical morphology will stimulate intestinal structure development and microbial community composition (8–10). For example, alfalfa hay can promote the development of intestinal epithelial villi; the feeding of high carbohydrate and low fiber feeding will be detrimental to the rumen development of calves; additives can directly or indirectly increase the proportion of probiotics (11–13). Research shows that intestinal microbial community can be directed to change, adjusting feeding patterns can help animals form beneficial microbial communities before weaning (14). This can not only promote the development of calf digestive ability, but also is indispensable for the development of intestinal immune function. Deng’s research on giant pandas has proved that *Streptococcus alactolyticus* can promote the dietary adaptation of giant pandas by participating in protein metabolism (15). Recent studies have also found that microbial communities also communicate biological information with the nervous system, it points to a new field: neuro microbiology, this further illustrates the huge impact of gut microbes on the host (16).

There is no fixed feeding mode for newborn calves. Appropriate feeding mode should be comprehensively formulated according to their nutritional needs, breed characteristics, feeding plan and other factors (17). For calves at this stage, growth is important, but more importantly, the healthy development of the digestive system based on the gastrointestinal tract. This will have a long-term impact on the subsequent growth and production of calves (18, 19). Therefore, it is very important to develop a suitable feeding mode. In this study, three different feeding modes were used to explore the effects of different feeding modes on the growth and development of ileum and blood metabolism of calves from the perspective of metagenomics and metabolomics. The relationship between intestinal development, digestive function and microbial community composition was explored to provide a theoretical basis for further improving the feeding pattern of calves. At the same time, it inspired us to explore the relationship between intestinal microorganisms and the nervous system from a new perspective of neurobiology.

## 2. Materials and methods

### 2.1. Ethics statement

This study was approved and conducted in accordance with the ethical standards of the Institutional Animal Care and Use Committee of the College of Animal Science and Technology, Sichuan Agricultural University, Sichuan, 611130, China.

### 2.2. Animals and feeding management

Nine 7-day-old healthy male Holstein calves with a body weight of about 41.6 kg (standard deviation = 0.563) and similar physical condition were selected. Raised in Sichuan Xuebao Dairy Group HonSFeng cattle farm. Nine calves were randomly divided into three groups, SH group: starter feed + hay + milk, SF group: starter feed + milk, TMR group: total mixed ration + milk. The feed composition and nutrient composition of the starter feed are shown in Table 1. The hay in the SH group was composed of alfalfa and oat grass in a ratio of 3:2, cut into 1.5 cm long, mixed with starter and fed. TMR feed (starter: alfalfa: oat grass: water = 0.30: 0.12: 0.08: 0.50), the starter was crushed into powder, the hay was cut to about 1 cm, and mixed with water to paste. Other feeding and management methods were carried out according to the existing methods of cattle farms, and calves were free to feed and drink water during the experimental period. Continuous feeding to 80 days of age, using electric shock bloodletting method to slaughter and record the slaughter weight.

**Table 1:** The feed composition and nutrient composition of the starter feed.

Component	Proportion (%)
Corn	61.16
Soybean meal	30.05
Compound vitamins	0.03
Trace elements	0.4
CaHCO3	0.67
CaCO3	1.65
50% choline chloride	0.1
Soybean oil	4.02
L-lysine	0.11
DL-methionine	0.43
Mineral additives	1.39
Nutritional ingredient	
NE,Mcal/kg	8.49
CP	17.73
Ca	3.10
Available phosphorus	0.79
Lys	0.99
Met	0

### 2.3. Serum sample collection and partial physiological index detection

After the calves were fasted for 24 h, the external jugular vein blood collection method was adopted (June 10, 2022), and 5 mL blood collection vessels (EDTAK2, Jiangsu Kangjian Medical Device Co., Ltd., Nanjing, China) were used to collect some calf blood, and heparin sodium was used for anticoagulation. Some blood samples were taken to determine blood routine indexes such as red blood cell count and white blood cell count (Chengdu Li Lai Biotechnology Co., Ltd., Chengdu). The blood samples were centrifuged at 4°C, 3000 r / min for 5 min to obtain the upper serum. Some serum samples were sent to Novogene Bioinformatics Technology Co., Ltd. (Beijing, China) for metabolomics analysis, and then some serum samples were selected to determine digestive and immune indexes such as α-amylase (C016-1-1), lysozyme (A050-1-1) and trypsin (A080-2; the kits were provided by Nanjing Jiancheng Bioengineering Institute).

### 2.4. Ileum morphological section analysis and sample collection

Slaughter of calves by electric bloodletting. The calves get up in the morning, use electric shock anesthesia after fasting blood collection, and then slaughter by carotid artery bloodletting. After slaughter, the contents of the ileum of the calf were quickly placed in a 2 mL frozen tube and stored in liquid nitrogen at −80°C. The unsealed ileum tissue was collected and fixed with 10% neutral formaldehyde solution. After dehydration, pruning, embedding, slicing, staining with hematoxylin and eosin (HE), sealing and other steps, the Pannoramic 250 digital slice scanner produced by Hungary 3DHISTECH company was used to collect images of the slices.

### 2.5. Microbial metagenomic sequencing and functional annotation analysis

According to the Tiangen Magnetic Bead Kit (Tiangen Biotech, Beijing, China) instructions, microbial DNA was extracted from ileum content samples, and the purity and integrity of DNA were detected by agarose gel electrophoresis. The qualified DNA samples were broken into fragments of about 350 bp by ultrasonic crushing instrument. After repair, purification, PCR amplification and other steps, the preliminary database was completed. Qubit2.0 was used for quantification, and then NEBNext ® Ultra DNA Library Prep Kit for Illumina (NEB, USA) was used to construct a metagenomic library and sequenced on the Illumina HiSeq Xten platform. The library construction and sequencing work was completed by Novogene Bioinformatics Technology Co., Ltd. (Beijing, China). The clean date was obtained by filtering the raw date obtained by sequencing, and the clean date of each sample was assembled and analyzed by MEGAHIT software (20, 21) (v1.0.4-beta^1^). Then MetaGeneMark software (22) (V2.10^2^) was used for gene prediction, and the genes predicted by each sample were put together to construct gene catalogue. Starting from the gene catalogue, the clean data of each sample was synthesized to obtain unigenes for subsequent analysis (23).

The obtained unigenes were compared with the NCBI’s NR database (24) (Version 2018-01-02^3^) to determine the species annotation information of each unigenes. Combined with the gene abundance table, the abundance information of each sample in phylum, genus and species was obtained. PCA (25) (RADE4 package, version 2.15.3) and LEfSe (26) (LDA score default to 3) analysis were used to compare the different species between groups. Finally, unigenes were compared with KEGG database (27, 28) (version 2018-01-01^4^), CAZy database (29) (version 201,801^5^) and eggNOG database (30) (version 4.5^6^) to obtain the relative abundance and functional annotation differences between groups (31).

### 2.6. Serum metabolomics analysis

The metabolites in the ileum were studied based on LC–MS technology. After preliminary treatment, the supernatant was injected into the ultra-performance liquid chromatography–tandem mass spectrometry (UHPLC–MS/MS) system for analysis (32). Firstly, the raw data of mass spectrometry were imported into Compound discoverer 3.1 software for spectral processing and database retrieval, and the qualitative and quantitative results of metabolites were obtained. Then, the quality of data was controlled to ensure the accuracy and reliability of the data. Using high-resolution mass spectrometry (HRMS) technology, we can make the non-target metabolic group as much as possible to detect the molecular characteristic peaks in the sample. The raw data after offline is preprocessed by CD3.1 data processing software. In order to make the identification accurate, we extract the peaks according to the set of ppm, signal-to-noise ratio (S/N), additive ions, and other information and quantify the peak area. Then mzCloud, mzVault, and MassList databases were compared to identify metabolites. Finally, metabolites with a coefficient of variation of less than 30% in QC samples were retained as the final result. The metabolites were compared with KEGG, HMDB, and other databases to obtain the annotation results. Then, a multivariate statistical analysis of metabolites was performed, including principal component analysis (PCA), partial least squares discriminant analysis (PLS-DA) to establish the relationship between the expression of metabolites and samples (33). According to the results of Q2 and R2, the model was judged to reveal the differences in metabolic patterns between different groups. KEGG enrichment pathway analysis was performed on the differential metabolites to obtain clearer and more detailed differential analysis results.

## 3. Results

### 3.1. HE staining section of ileum tissue

**Figure 1:**
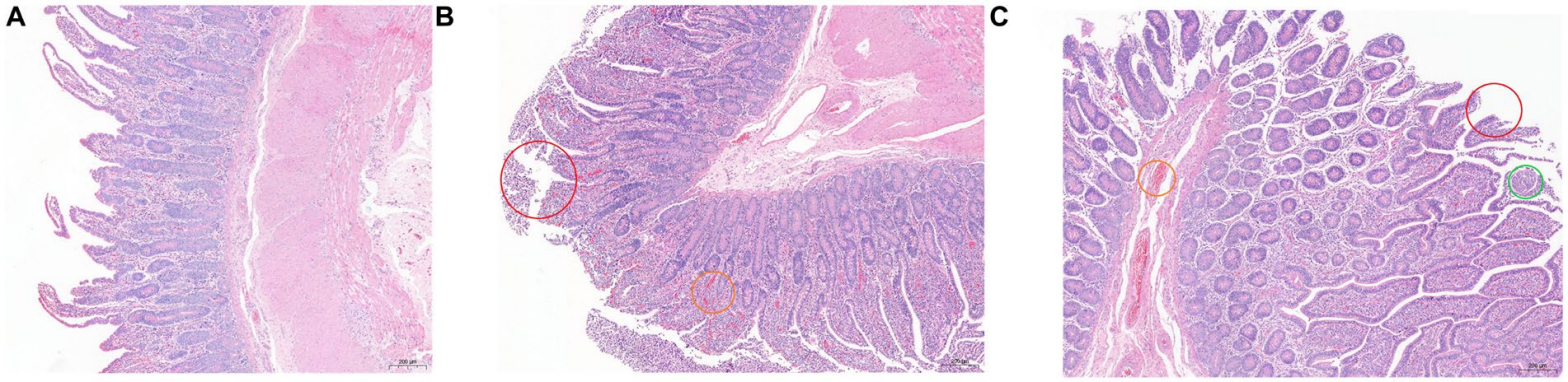
Hematoxylin and eosin (100×) -stained ileal tissue samples of Holstein calves fed different diets. **(A)** SH group; **(B)** SF group; **(C)** TMR group. Red circle marked mucosal epithelial shedding; the orange circle marked congestion; green circle marked as parasitic lesions.

The results of HE staining are shown in Figure 1. Compared with the other two groups, the epithelial villi in the SH group were relatively complete, the intestinal epithelial villi were high and well developed, the goblet cells were closely arranged, and some inflammatory cells were seen in the lamina propria. There were a large number of epithelial mucosal shedding in the SF group, and the inflammatory cells in the lamina propria were also more than those in the SH group, accompanied by congestion; the mucosal integrity of the TMR group was the worst, accompanied by inflammatory cells and congestion, and even obvious parasitic infection was observed in some sections.

### 3.2. Daily weight gain and some physiological indexes test results

In this experiment, 9 newborn, healthy and healthy male calves with no significant difference in birth weight were selected. The birth weight was about 41 kg. When the calves were slaughtered at 80 days of age, there were significant differences in weight among the three groups (*p* = 0.002). The average weight of SH group and SF group TMR group was 100.62 ± 1.94, 100.07 ± 1.92 and 90.77 ± 2.42. And the daily weight gain was 0.745 kg / day, 0.740 kg / day and 0.604 kg / day, respectively.

**Figure 2:**
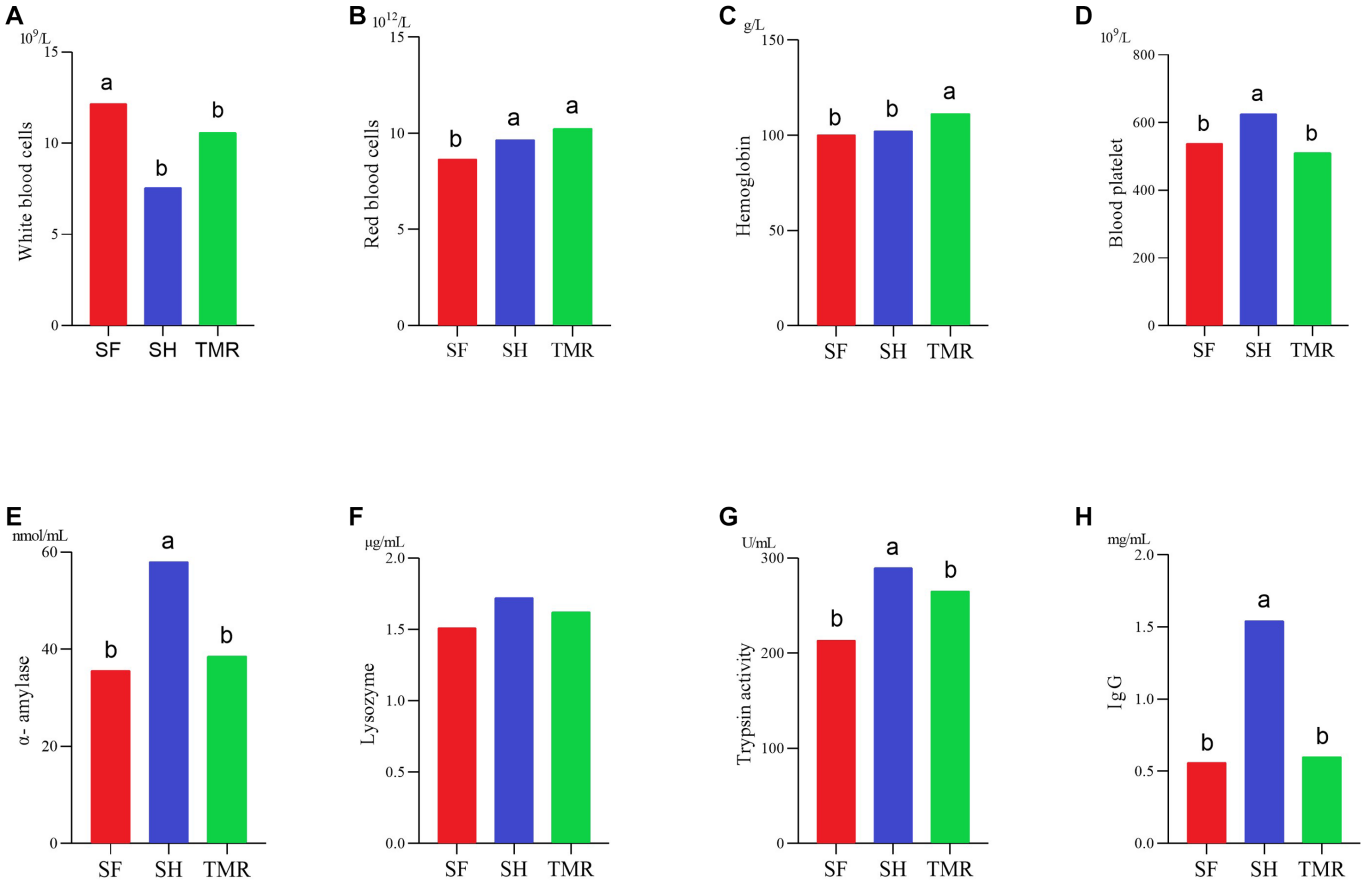
Some physiological indexes test results. **(A–D)** It’s blood routine determination results. **(E–H)** It’s digestive enzyme and immunoassay results.

In Figure 2, some blood routine data. The results showed that the number of white blood cells SH was significantly less than that of SF group and TMR group, which may be due to the more severe inflammatory response in SF group and TMR group. The number of red blood cells in the TMR group was the highest. The hemoglobin content in the TMR group was also the highest in the three groups. The results of digestive enzymes and immunity showed that the contents of amylase, trypsin and lysozyme in SH were the highest in the three groups. The IgG content of SH group was also the highest in the three groups. These indicators show that the digestive ability and immune ability of SH group are higher than the other two groups.

### 3.3. Ileum microbial metagenomic data processing

As shown in Table 2, a total of 112,964.83 raw data were measured in 9 samples, and 112,791.07 clean data were obtained after Illumina pretreatment and filtering, with an average CG content of 45.87% and an effective value of more than 99.85%. The average length of scaftigs of 9 samples was 1434.85.

**Table 2:** Preprocessing of sequencing data.

Sample ID	Raw date	Clean date	Clean_GC(%)	Effective (%)	Total Len.	Num	Average Len.
SH	12,203.11	12,190.28	50.27	99.895	161,322,099	112,565	1,433.15
	12,496.78	12,475.11	47.34	99.827	217,508,616	134,445	1,617.83
	12,651.70	12,609.24	43.09	99.664	153,544,642	102,299	1,500.94
SF	12,451.10	12,439.42	50.13	99.906	156,100,365	94,575	1,650.55
	12,084.00	12,062.04	47.41	99.818	1,778,426	2,347	757.74
	12,573.19	12,546.90	38.51	99.791	177,806,423	124,453	1,428.70
TMR	13,345.45	13,323.13	44.12	99.833	344,856,565	217,392	1,586.34
	12,432.55	12,423.68	50.65	99.929	297,219,715	189,565	1,567.90
	12,726.95	12,721.27	41.27	99.955	177,848,979	129,771	1,370.48

### 3.4. Analysis of correlation between groups and differences in microbial community

**Figure 3:**
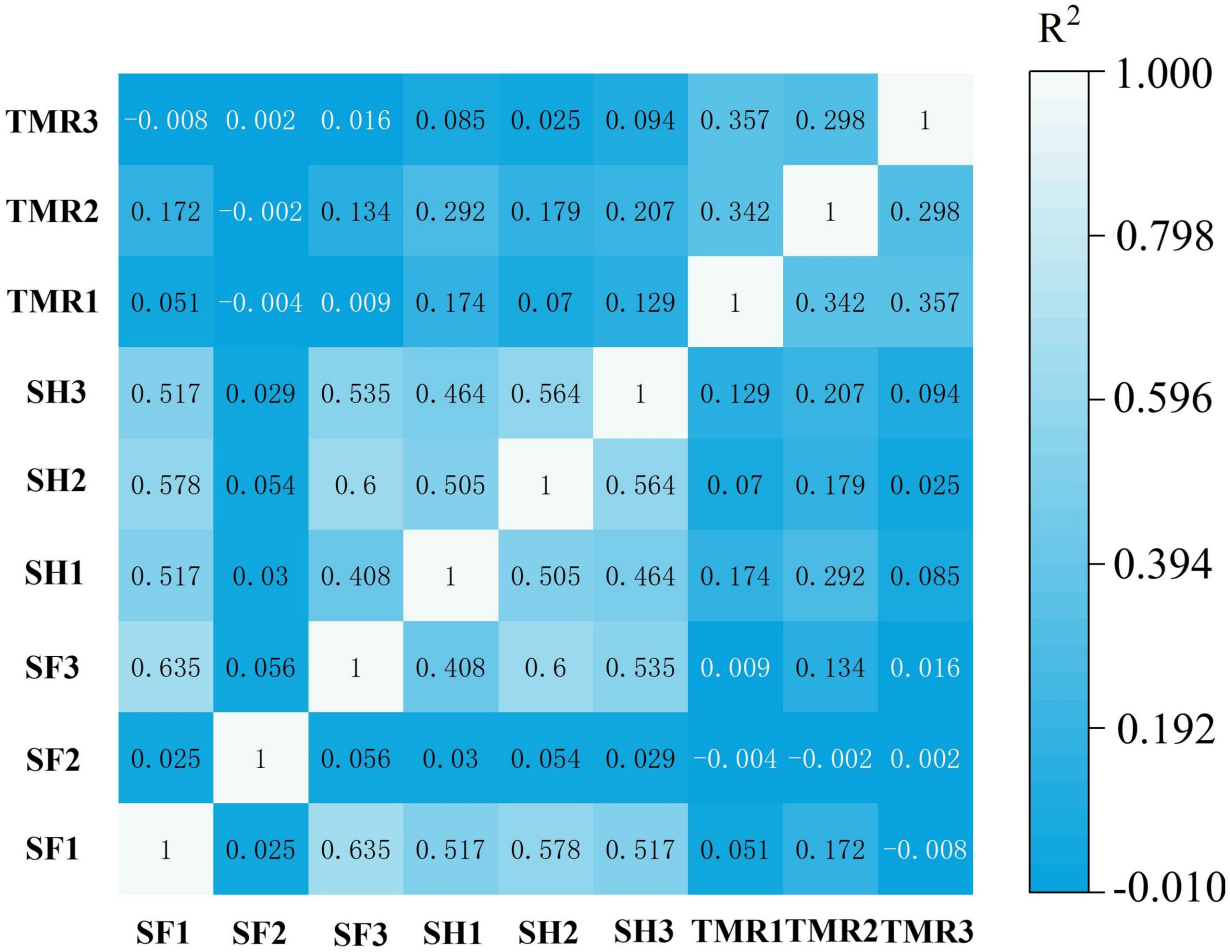
Correlation analysis between groups of samples.

Based on the number of genes, correlation analysis was performed on 8 samples. The results are shown in Figure 3. The correlation between SF group and SH group was high, but the correlation between TMR group and SF group and SH group was low.

**Figure 4:**
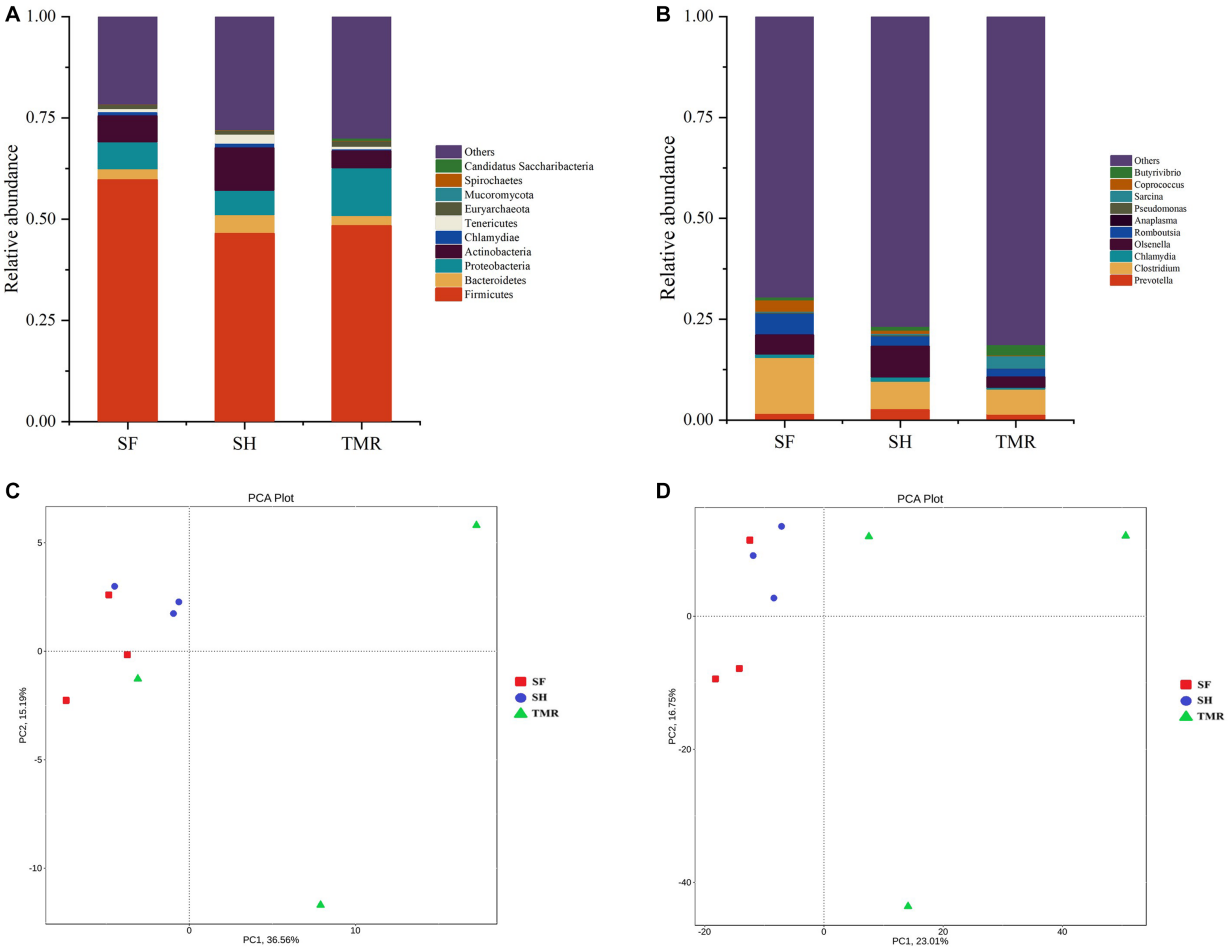
Analysis of microbial community differences between groups. **(A)** Relative abundance of species at phylum level TOP10. **(B)** Relative abundance of genus level species TOP10. **(C)** Phylum level species PCA analysis. **(D)** PCA analysis of genus-level species.

The processed genes were compared with the database to obtain the species annotation information of each sequence and compared after classification at each level. The results are shown in Figure 4. The dominant bacteria in the three groups were Firmicutes under the phylum level classification. The subdominant bacteria in group SF were *Proteobacteria* and *Chlamydiae*. The subdominant flora in SH group was *Actinobacteria*, more than the other two groups. The subdominant flora in the TMR group was *Proteobacteria*. Under the genus level classification, the dominant flora of group SF was *Clostridium* and *Chlamydia*; the dominant flora in SH group was *Olsenella*, which was more than that in TMR group and SF group, and the secondary dominant flora was *Clostridium*. The dominant flora in the TMR group was *Clostridium* secondary dominant flora *Sarcina* and more than that in the SF and SH groups.

**Figure 5:**
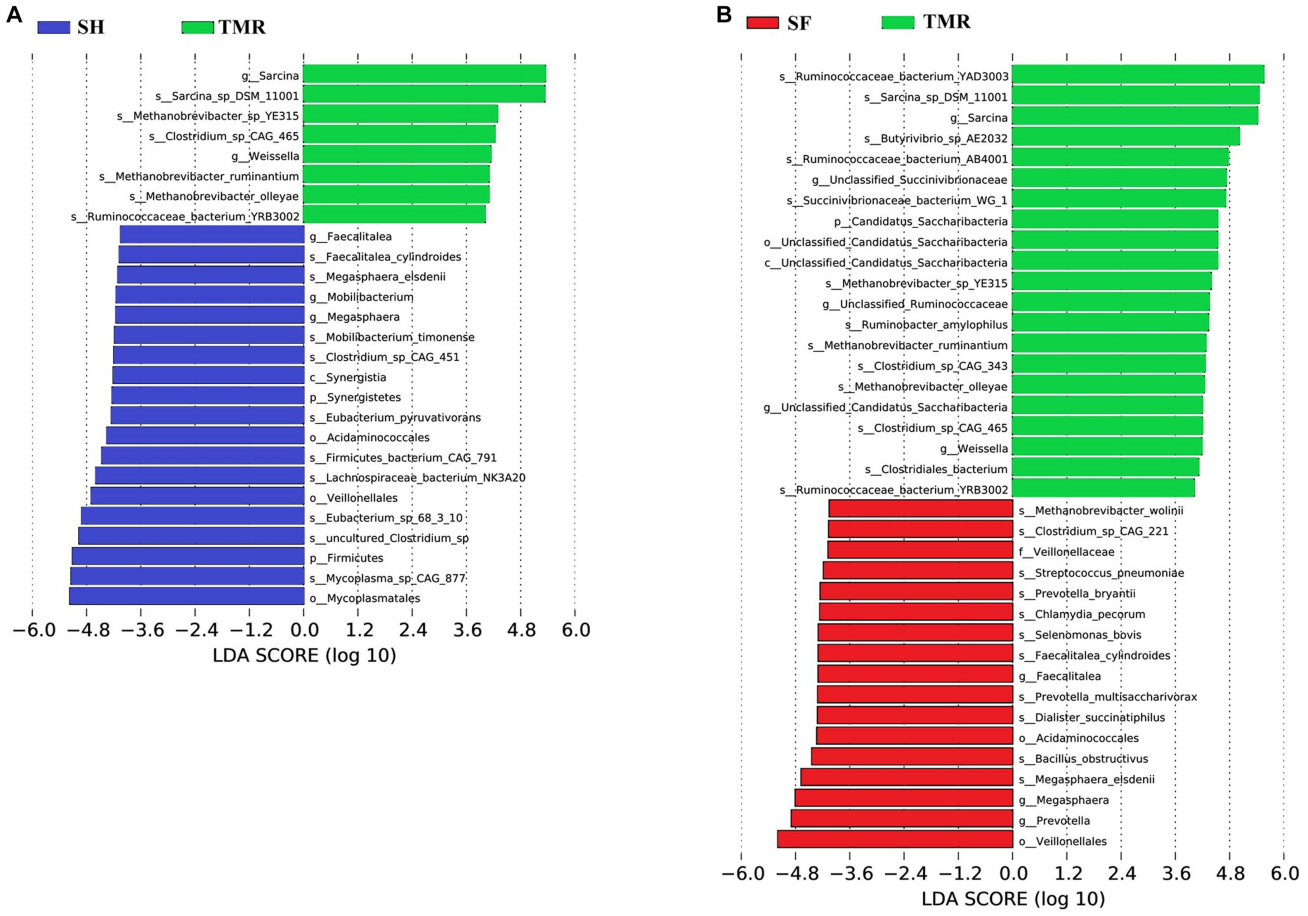
LDA score of LEfSe-PICRUSt. **(A)** LDA score analysis diagram of different species between SH group and TMR group. **(B)** LDA score analysis diagram of different species between SF group and TMR group.

The results of PCA analysis showed that under the classification of phylum level and genus level, the intra-group difference of SH group was the smallest, the intra-group difference of SF group was slightly larger than that of SH group, and the intra-group difference of TMR group was the largest. There were significant differences among the three groups, and the intra-group difference of TMR was greater than the inter-group difference, which indicate that after feeding the total mixture, the ileum microbes of calves were greatly different. In order to screen species with significant differences between groups, the results of LDA analysis (Figure 5) showed that the main differences between SH group and TMR group were p _ Firmicutes and o _ *Mycoplasmatales* in SH group and p _ *Sarcina* in TMR group. The main differential flora between SF group and TMR group were o _ *Veillonellaceae*, g _ *Prevotella* in SF group and p _ *Sarcina* in TMR group.

### 3.5. Functional analysis of differential species

**Figure 6:**
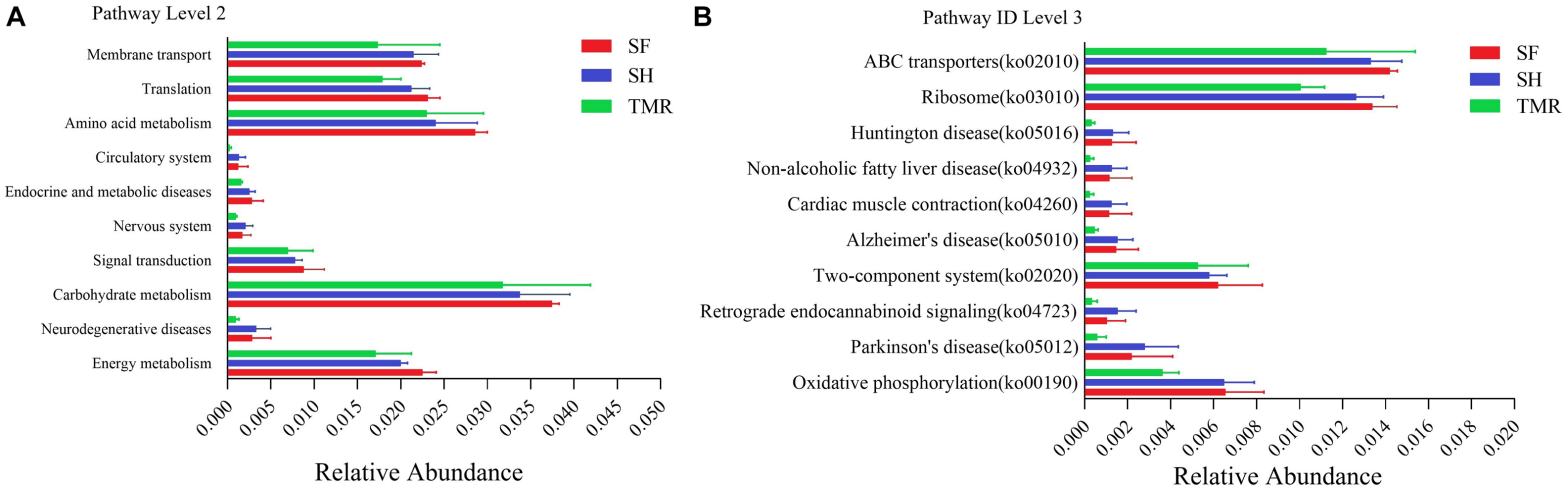
KEGG pathway enrichment analysis. **(A)** KEGG level 2 enrichment analysis top 10 between groups. **(B)** KEGG level 3 enrichment analysis top 10 between groups.

The relative abundance of KEGG pathway enrichment in the three groups was analyzed (Figure 6). It was found that among the 45 pathways under the second level classification, the SH group was mainly enriched in carbohydrate metabolism, followed by amino acid metabolism, translation and membrane transport; the dominant pathways in the TMR group were carbohydrate metabolism and amino acid metabolism. It is worth noting that the TMR group was enriched in cell growth and death and drug resistance: antimicrobial pathway higher than SH group and SF group. These genes were further compared in the metabolic pathways of the third level, and were enriched in 302 pathways. The three groups all enriched more genes in ABC transporters (pathway ID: ko 02010), ribosome (pathway ID: ko03010), purine metabolism (pathway ID: ko 00230) and pyrimidine metabolism (pathway ID: ko00240), but the SF group was more and the TMR group was the least. Interestingly, the number of genes enriched in Cell cycle-Caulobacter (pathway ID: ko04112), necroptosis (pathway ID: ko04217) and other pathways under the classification of cell growth and death pathway was the least in SH group, and more in TMR group and SF group. The Cationic antimicrobial peptide (CAMP) resistance (pathway ID: ko01503) under the Drug resistance: Antimicrobial classification was similar.

**Figure 7:**
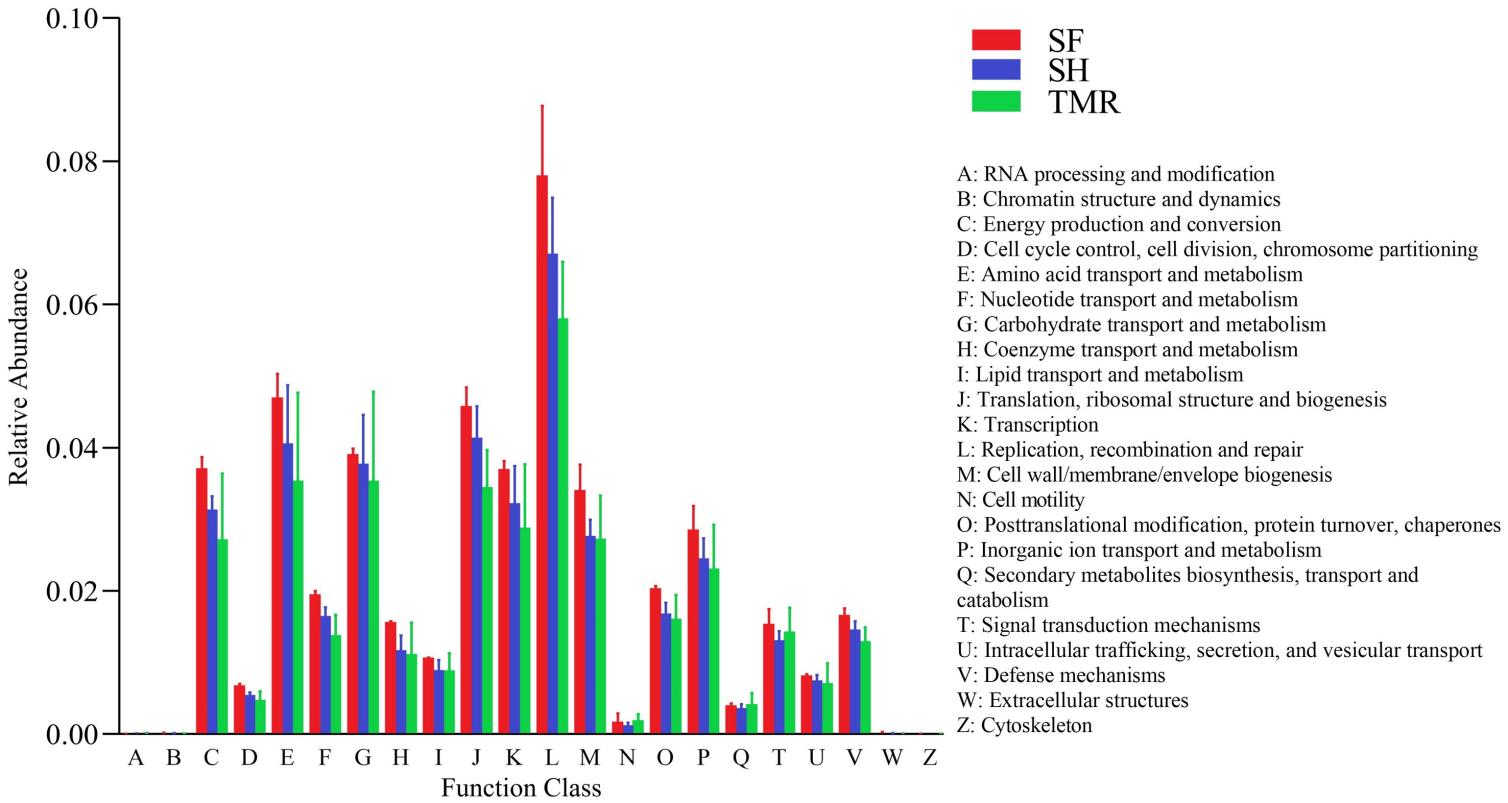
EggNOG enrichment analysis.

The results of EggNOG analysis (Figure 7) showed that the most abundant genes were enriched in Replication, recombination and repair, followed by Amino acid transport and metabolism and Translation, ribosomal structure and biogenesis. After comparing the relative abundance of genes annotated in eggNOG in the three groups, it was found that the main enrichment pathways of the three groups were similar, with the most in SF group and the least in TMR group. The relative abundance of secondary metabolites biosynthesis, transport and catabolism, Cell motility and RNA processing and modification in TMR group was higher than that in the other two groups.

**Figure 8:**
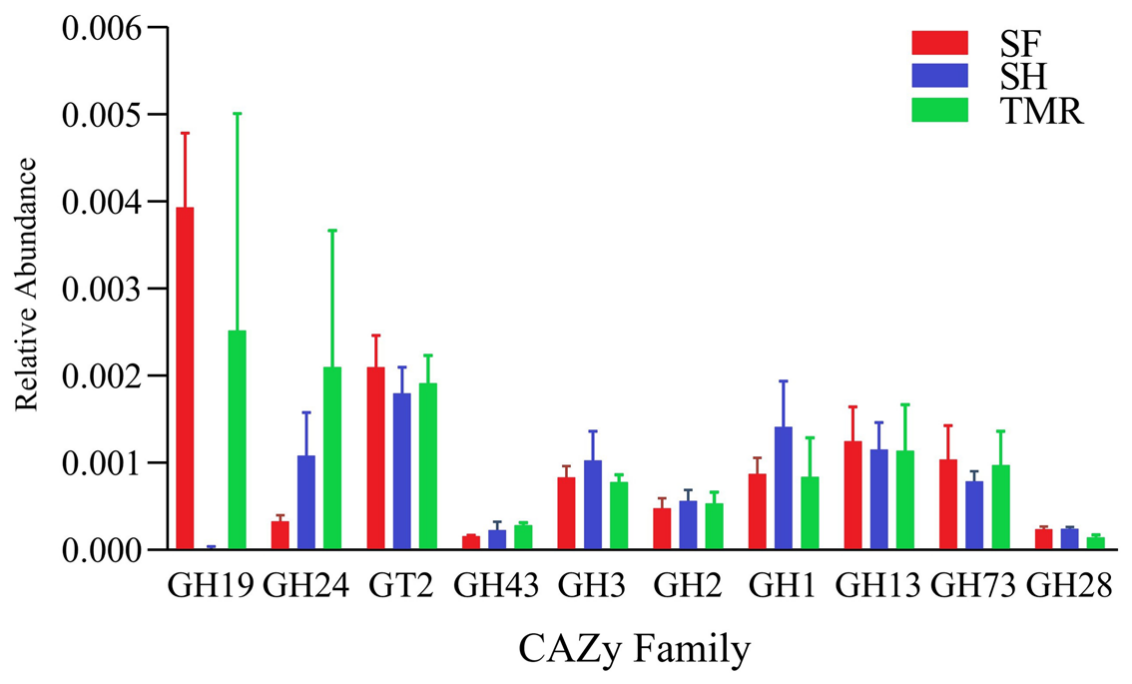
CAZy enrichment analysis top 10 between groups.

After comparing the relative abundance of CAZy genes in the three groups (Figure 8), it was found that Glycoside Hydrolases (GH) were the main ones, and there were differences in CAZy family under the second level classification. The CAZy family of SH group was more evenly distributed, and the most enriched families were GT2 family and GH1 family, among which GH1, GH3 and GH25 family were higher than the other two groups. GH19, GT2 and GH13 family were more enriched in group SF than in the other two groups. There were more GH19, GH24 and GT2 family in TMR group, and GH24 was more than the other two groups. The CAZy families that attract our attention are GH1 and GH19. The differences between the three groups are also mainly reflected in these two families. The enrichment of GH1 in SH group was more than that in SF group and TMR group, mainly due to the β-glucosidase (EC 3.2.1.21) of GH1 family under level 3 classification. GH19 was more enriched in SF group and TMR group than in SH group, mainly chitinase (EC 3.2.1.14) of GH19 family under level 3 classification.

### 3.6. Analysis of serum differential metabolites

**Figure 9:**
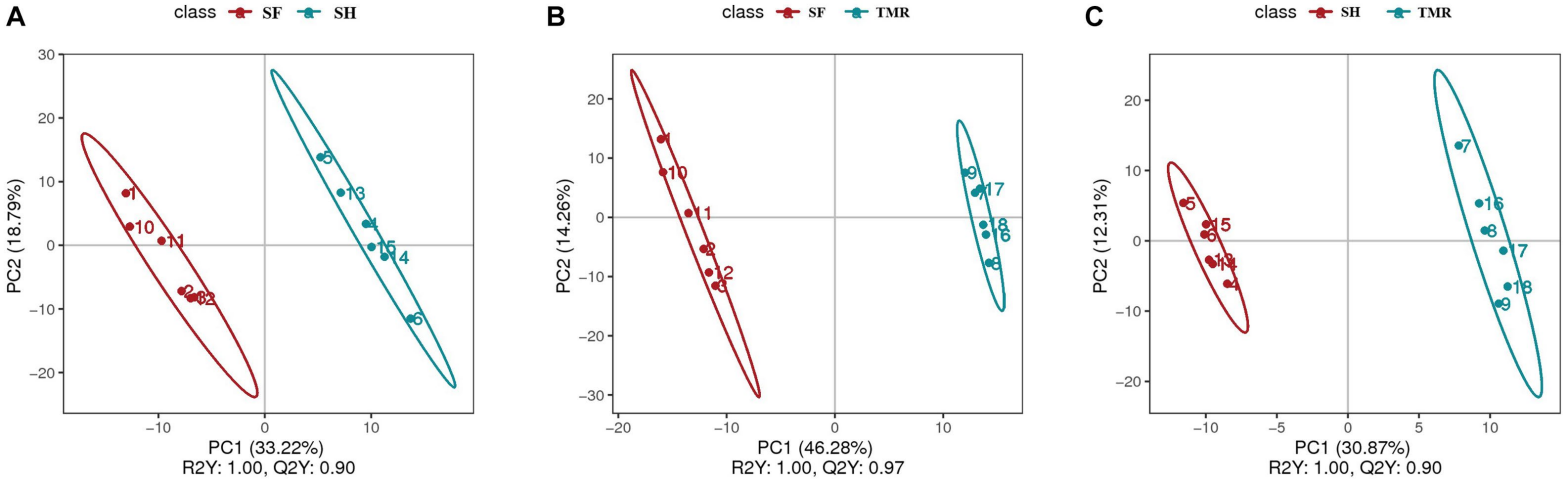
PLS-DA analysis of serum metabolites between groups. **(A)** PLS-DA analysis of SF group and SH group; **(B)** PLS-DA analysis of SF group and TMR group; **(C)** PLS-DA analysis of SH group and TMR group.

After analyzing the blood metabolites of the three groups, a total of 403 cathode metabolites were found. PLS-DA analysis (Figure 9) of these metabolites between groups showed that the separation between groups was obvious, the degree of polymerization in the group was high, and the model parameters of each group also met the standard, indicating that the results were stable and reliable. Further analysis and screening of differential metabolites (Table 3) showed that there were 87 differential metabolites in the cathode of SF group and SH group, 17 were significantly up-regulated and 70 were significantly down-regulated. There were 152 differential metabolites in the cathode of SF group and TMR group, 27 were significantly up-regulated and 125 were significantly down-regulated. There were 108 differential metabolites in the cathode of SH group and TMR group, 26 were significantly up-regulated and 82 were significantly down-regulated.

**Table 3:** Number of differential metabolites in serum.

Compared samples	N total identified	N Signif. different	N Signif. up	N Signif. down
SF vs. SH	403	87	17	90
SF vs. TMR	403	108	26	82
SH vs. TMR	403	152	27	125

N Total Identified, Total number of differential metabolites. N Signif. Different, Number of significant differential metabolites. N Signif. Up, Number of significantly higher metabolite contents. N Signif. Down, Number of significantly lower metabolite.

### 3.7. KEGG enrichment analysis of differential metabolites

The selected differential metabolites were subjected to KEGG enrichment analysis to determine the effects of differential metabolites on key blood metabolic pathways. The results showed that the differential metabolites between SF group and SH group were mainly enriched in cortisol synthesis and secretion (value of *p* = 0.032). The differential metabolites between SF group and TMR group were mainly enriched in cAMP signaling pathway (value of *p* = 0.043), dopaminergic synapse (value of *p* = 0.043), Taste transduction (value of *p* = 0.043). The differential metabolites between SH group and TMR group were mainly enriched in cAMP signaling pathway (value of *p* = 0.024), dopaminergic synapse (value of *p* = 0.024), taste transduction (value of *p* = 0.024), tyrosine metabolism (value of *p* = 0.046). The specific pathway information and differential metabolites enriched in the pathway are shown in the Table 4.

**Table 4:** KEGG enrichment analysis results of differential metabolites.

	Map ID	Map title	Value of *p*	*N*	Meta IDs
SF vs. SH	map04927	Cortisol synthesis and secretion	0.03241	81	Pregnenolone^##^; Cortodoxone**
	map04934	Cushing’s syndrome	0.03241	81	Pregnenolone^##^; Cortodoxone**
	map00140	Steroid hormone biosynthesis	0.04173	81	Pregnenolone^##^; Androsterone glucuronide*; Cortodoxone**
SF vs. TMR	map04024	cAMP signaling pathway	0.04283	81	Dopamine^##^; Noradrenaline^##^; Adenosine 5′-monophosphate^##^
	map04728	Dopaminergic synapse	0.04283	81	Dopamine^##^; Levodopa^##^; 3-Methoxytyramine^##^
	map04742	Taste transduction	0.04283	81	Noradrenaline^##^; Saccharin**; Adenosine 5′-monophosphate^#^
SH vs. TMR	map04024	cAMP signaling pathway	0.02372	81	Dopamine^##^; Noradrenaline^##^; Adenosine 5′-monophosphate^#^
	map04728	Dopaminergic synapse	0.02372	81	Dopamine^##^; Levodopa^##^; 3-Methoxytyramine^#^
	map04742	Taste transduction	0.02372	81	Noradrenaline^##^; Saccharin**; Adenosine 5′-monophosphate^#^
	map00350	Tyrosine metabolism	0.04598	81	Dopamine^##^; Levodopa^##^; Noradrenaline^##^; Tyrosol**; 3-Methoxytyramine^#^

In the Meta IDs annotation, ^#^ represents a significant down-regulation, ^##^ represents a significant down-regulation, ^*^ represents a significant up-regulation, ^**^ represents a significant up-regulation.

## 4. Discussion

The ileum is located in the posterior part of the small intestine and is the main place for digestion and absorption of nutrients. Therefore, the development of ileal function is essential for the health and growth of calves. We observed from the sections after HE staining that the intestinal epithelial villi in the SF and SH groups were better developed, the villus height was higher, and the goblet cells in the villi were more closely arranged. The goblet cells can secrete mucus and form a physical barrier on the surface of the intestinal mucosa, which is the main component of the intestinal barrier (34). These characteristics mean that the ileum of SF group and SH group has a larger absorption and digestion area, and the intestinal morphology is more conducive to the absorption of nutrients, and has a stronger barrier function, which is the characteristic of healthy development of calf intestine, which is conducive to the development of digestive ability and gastrointestinal health of calves (35, 36). The research of Ma showed that the intestinal tract of calves must undergo development, physical and physiological changes, before they can better adapt to the transition from milk to solid feed (37). Studies have shown that the healthy development of intestinal epithelial environment is conducive to the development of intestinal probiotics and the stability of microbial communities (38). We observed a more obvious inflammatory response in the TMR group, and the intestinal wall in the SH group was relatively complete. After subsequent analysis, we believe that it may be due to the digestive ability of calves and maturity, which cannot adapt to the inflammatory response caused by TMR feed. The parasite phenomenon may be caused by the invasion of pathogens. Whether it is related to the total mixed feed needs further study.

Metagenomic analysis compared the microbial community differences among the three groups. The results showed that SH group was enriched in *Actinobacteria* and more than the other two groups. In previous studies, except for a small number of pathogenic bacteria, *Actinobacteria* is usually a probiotic that is conducive to the digestion and absorption of organic matter. In some farms, probiotics are used to selectively stimulate the growth of *Actinobacteria* (39). Moreover, *Actinobacteria* can also produce secondary metabolites such as natural drugs, enzymes, and active factors, and is considered to be a potential probiotic (40). Bifidobacterium under the classification of *Actinobacteria* plays an important role in regulating the immune system and preventing intestinal diseases. It can reduce the inflammatory response by producing short-chain fatty acids and regulating inflammatory factors. The proliferation of *Actinobacteria* may be the key to regulating immunity and reducing inflammatory response in SH group. Some studies have shown that *Actinobacteria* also plays a role in reducing pathogens such as Escherichia-Shigella, indicating that it can also have a positive effect on preventing diarrhea (41). *Olsenella* has obvious advantages in SH enrichment. *Olsenella* belongs to probiotics under the classification of lactic acid bacteria. Studies have shown that *Olsenella* is positively correlated with butyrate. Butyrate is one of the main volatile fatty acids produced by rumen fermentation, and ileum is also the main part of absorbing butyrate. Butyrate can provide energy for *Olsenella* and can strengthen the intestinal barrier function, which is conducive to intestinal health (42). The study of Zhang also showed that individuals with higher relative abundance of *Olsenella* have a stronger ability to produce short-chain fatty acids, which can effectively affect the structure of the intestinal epithelial mucus layer, stimulate the development of intestinal epithelial tissue, nourish the development of intestinal epithelial cells such as goblet cells, and reduce the risk of inflammatory bowel disease in individuals (43).

The dominant bacteria in the SF group were *Chlamydiae* and *Proteobacteria*. *Chlamydiae* is a pathogen that may cause enteritis (44, 45), but no diarrhea symptoms were found in this individual, and there may be a case of invisible infection of *Chlamydiae* (46). At the same time, other pathogens with high correlation with *Chlamydiae*, such as Campylobacter, were not found. *Chlamydiae*, as a potential pathogen, may be related to the local inflammation observed in the SF group, which may lead to impaired intestinal absorption and induce other diseases (47). Timely treatment is needed. The study of Xia in weaned piglets showed that the addition of plant essential oils containing substances such as tributyrin to the feed can resist Chlamydia, regulate microbial morphology, and improve intestinal villus morphology (48).

*Proteobacteria* in both TMR group and SF group have high abundance. Previous studies have shown that many intestinal pathogenic bacteria belong to *Proteobacteria*, and the relative abundance of *Proteobacteria* is too high, which may cause inflammation and cause calf diarrhea (49, 50). Shin used the enrichment of *Proteobacteria* as one of the important markers of intestinal microflora imbalance in healthy individuals. In general, *Proteobacteria* will not be significantly enriched in healthy individuals (51). The study of Han on weaned piglets showed that *Proteobacteria* could activate inflammation-related cytokines and cause intestinal inflammation damage. At the same time, the daily weight gain of piglets with enrichment of *Proteobacteria* was significantly reduced, which indicated that the intestinal nutrition absorption function was also affected (52). The study of Varada on newborn buffaloes found that feeding probiotics can regulate the composition of intestinal microbial communities by reducing the abundance of *Proteobacteria* (53). Numerous studies have shown that the enrichment of *Proteobacteria* can lead to inflammatory response and cause intestinal epithelial cell dysfunction (54, 55). This may be one of the reasons why the TMR group had a large number of inflammatory cell infiltration, intestinal epithelial cell shedding, and even diarrhea symptoms. *Sarcina* enriched in TMR is a pathogen that has been reported many times. Katharine found that *Sarcina* was associated with gastric ulcer and fungal infection in calves and goats, which may be the cause of gastritis and enteritis (56). The study of Simpson also found that *Sarcina* may cause necrosis of the intestinal epithelial surface and may be related to parasitic infection (57). Many studies have pointed out that *Sarcina* is associated with diarrhea, inflammation and other symptoms. *Sarcina* is significantly enriched in the intestinal tract of horses parasitized by ascaris, and *Sarcina* is often found in goats, rabbits and other animals with diarrhea symptoms or fungal infection (58, 59). The study of Zhuo on piglets directly pointed out that the enrichment of *Sarcina* is not conducive to the healthy development of the intestinal tract, affects the production performance and feed absorption rate, and increases the probability of intestinal pathogen infection. They also pointed out that *Sarcina* has a great relationship with the reduction of microorganisms that produce short-chain fatty acids and feeding patterns (60). This is similar to our findings. Therefore, we believe that the inflammatory response, parasitic infection and diarrhea symptoms in the TMR group are closely related to *Sarcina*.

After comparing the enrichment of KEGG signaling pathway, we found that in Cell cycle-Caulobacter (pathway ID: ko04112) and Necroptosis (pathway ID: ko04217) signaling pathways, SH group was less, SF group and TMR group were more. Cell cycle-Caulobacter is a key pathway that can regulate DNA replication, cell cycle and cell topology. Cell cycle response regulator is the core of his network. Cell cycle-Caulobacter regulates the asymmetric division of bacterial cells, resulting in stalked cells and swarmer cells with different fates (61). The stalked type of progeny cell type will enter the S phase, while the swarmer type will remain in the G1 phase (62). We compared the metabolites annotated in the three groups, and found that the unique metabolites in the SF group and the TMR group eventually pointed to polar morphogenesis and pili biogenesis, while the unique metabolites in the SH group pointed to flagellar ejection holdfast biogenesis, stalk formation, that is, the cells in the SF group, TMR group and SH group went to different divisions, and different daughter cells had different cell membrane structures and cell morphology. This leads to differences in subsequent functions (63, 64). Studies have shown that phages with pili and flagella structures are more susceptible to the host through CtrA regulation (65). This molecular network regulation has a direct relationship with bacterial infection. McAdams ‘s study pointed out that the function within the Cell cycle-Caulobacter network is conserved, but after asymmetric division, due to differences in cell function and protein, downstream specific coupling species will be very different (66). Therefore, we boldly speculate that the Cell cycle-Caulobacter pathway in the SH group is not as active as the other two groups, and the final cell division results are different, which may be one of the important reasons for the differences in bacterial infection and inflammatory response between the SH group and the TMR group and the SF group. The difference in Necroptosis is clearer. SH has no unique metabolites, while the unique metabolites of SH group and TMR group are the same, which are H2AX, VDAC, Drp1, ESCRT-III. These metabolites directly or indirectly point to inflammatory response, mitochondrial damage, etc., and their activity may lead to cell damage, and ultimately necroptosis (67). Yi’s study found that plasma pathogens such as *Staphylococcus aureus* can lead to cell necroptosis by affecting mitochondrial membrane potential and membrane permeability. This necroptosis is regulated by calmodulin-dependent protein kinase II (CaMKII) and mixed lineage kinase domain-like (MLKL) (68). The unique metabolites we found in TMR group and SF group were involved in the above reaction process. Huang’s study also pointed out that activation of MLKL and CaMKII can lead to mitochondrial dysfunction and pro-inflammatory response (69). We also found that the unique metabolites of TMR group and SF group were concentrated in cytochrome c oxidase \ reductase after comparing the Oxidative phosphorylation (pathway ID: ko00190) of the three groups. We believe that this may be due to bacterial infection that activates pathways such as MLKL, which leads to necroptosis caused by mitochondrial dysfunction and promotes inflammatory response, and ultimately leads to damage to intestinal epithelial structure (70). In addition, intestinal flora may also interact with metabolic intermediates in Oxidative phosphorylation. Existing studies have found that NAD (nicotinamide adenine dinucleotide) can promote the secretion of intestinal mucin 2 (MUC2), enhance the ability of antibacterial infection, and alleviate intestinal inflammation by inhibiting NF-kB. Oxidative phosphorylation abnormalities and mitochondrial damage are likely to affect the levels of metabolites such as NAD, increasing the risk of intestinal inflammation and bacterial infection (71).

**Figure 10:**
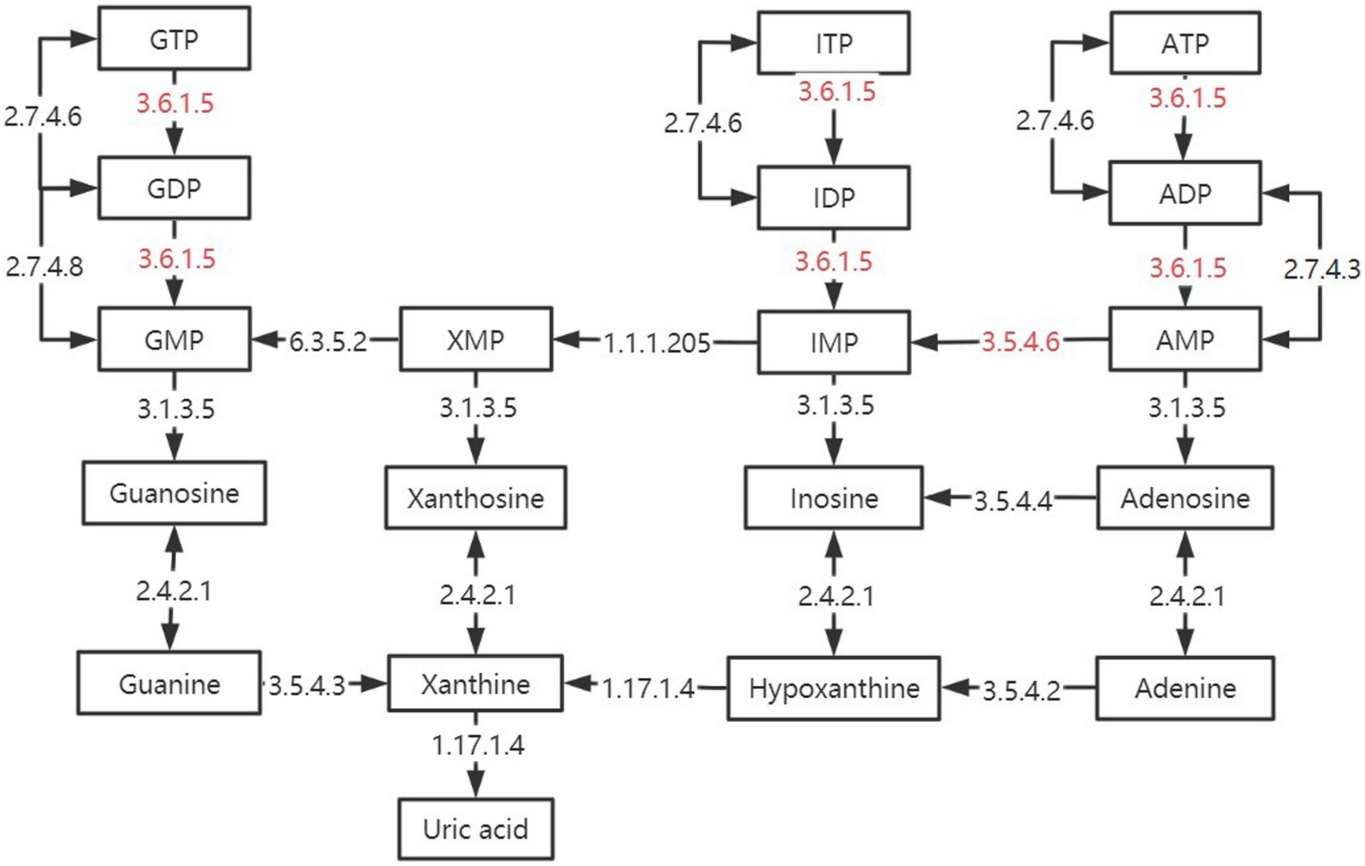
Differences in purine metabolic pathways between groups. The black box is marked as the intermediate product in the purine metabolic pathway. The black arrow is marked with enzymes annotated in the kegg enzyme database. The red-labeled enzyme is unique in SF and TMR.

In the two signaling pathways of Purine metabolism (pathway ID: ko 00230) and Pyrimidine metabolism (pathway ID: ko00240), the three groups have more enrichment, but their metabolic process is different. As shown in Figure 10, some of the reactions in the TMR and SF groups were more active than SH in these two pathways, including several key enzymes that catalyze one-way reactions, such as apyrase (EC: 3.6.1.5) and AMP deaminase (EC: 3.5.4.6), which are unique to the SF and TMR groups. Studies have shown that when the body is in an inflammatory or stress state, the demand for nucleotides will increase to promote intestinal cell proliferation and tissue repair (72, 73). The activity of apyrase (EC: 3.6.1.5) will lead to excessive metabolic intermediates such as GMP and AMP. Their final products all point to uric acid, but the enzyme for the next metabolism of uric acid is not active. Although nucleotide metabolism in the SF and TMR groups may be an autonomous repair of inflammation, it also increases the risk of accumulation of substances such as uric acid. In general, purine catabolism produces metabolites with potential toxicity (74, 75). Excessive accumulation of uric acid may lead to the formation of sodium salt deposition in shutdown, skin and other parts, causing pain. It may also cause kidney damage and lead to the occurrence of uric acidemia, which is common in long-term high protein intake of livestock and poultry (76, 77); in serious cases, it may also lead to atherosclerosis (78). Apyrase is involved in the dephosphorylation of GTP, ATP, UTP, CTP and other substances, and is a one-way reaction. ATP, GTP and ITP were dephosphorylated to produce Uric acid. UTP and CTP were also dephosphorylated to produce Uracil, which was converted into β-Alanine to participate in the next reaction. After dephosphorylation, dTTP is dephosphorylated to generate dTMP, which will further generate Thymine, and then generate (R)-3-Amino-2-methylpropanoate to participate in Valine, leucine and isoleucine degradation pathway. Therefore, we inferred that the Nucleotide metabolism of the three groups was more enriched because the intestine of the calves was in the developmental stage, and the metabolism was vigorous because it responded to stimuli such as feed differences and microbial flora changes. However, the SF group and the SH group showed uniqueness in some metabolic processes due to the need for anti-inflammatory and epithelial cell repair. The activity of enzymes such as apyrase (EC: 3.6.1.5), and the enzymes not annotated to uric acid catabolism may lead to the risk of uric acid accumulation in the SF group and the TMR group.

By comparing the analysis results of the three groups of CAZy databases. The differences are mainly reflected in the GH1 family and the GH19 family. Under level 3 classification, the enrichment of β-glucosidase (EC 3.2.1.21) belonging to GH1 family in SH group was more than that in the other two groups. β-glucosidase mainly plays a role in glycolipid metabolism and is a key enzyme that catalyzes cellulose to produce glucose. We believe that the higher β-glucosidase activity in the SH group is closely related to its microbial community. Studies have shown that adding β-glucosidase to feed or activating β-glucosidase through diet can improve the digestive capacity of the gastrointestinal tract and benefit animal growth (79). β-glucosidase can also increase the activity of intestinal amylase (80). This is also consistent with our found, the amylase content of SH group was the highest. Zhang ‘s study found that castration changed the cecal digestion environment of dairy cows, significantly reduced the activity of β-glucosidase, reduced the relative abundance of Ruminococcaceae and other bacteria related to β-glucosidase, and reduced the daily gain of dairy cows (81). Therefore, the enrichment of β-glucosidase has a positive effect on calves. The chitinase (EC 3.2.1.14) in GH19 family showed more obvious differences. The enrichment of chitinase in SH group was much less than that in SF group and TMR group. Chitinase has strong specificity and can cleave chitin glycosidic bonds. Chitin is a component of invertebrates, arthropods, fungi, etc., but mammals can hardly synthesize chitin themselves. Therefore, attention should be paid when excessive chitinase is enriched in mammals, and it may be food containing chitin or microbial pathogens invade the body (82). Recent studies have shown that chitin protein is related to gastrointestinal diseases. Chitinase can play an important role in gastrointestinal digestion and diseases due to its specificity, and it is likely to become a new idea for the treatment of gastrointestinal diseases (83, 84). The study of Beier found that microorganisms can also produce chitinase, including Firmicutes, *Actinobacteria*, etc. Chitinase can specifically hydrolyze chitin and make it lose pathogenicity, which is particularly critical for mammals (85). We observed local inflammatory reactions in both the SF group and the TMR group. Parasites also appeared in the TMR group. It is most likely that foreign pathogens produce chitin, which stimulates some of the bacteria in the intestine that can release chitinase to respond, causing differences in intestinal flora.

In addition to this, there is a more critical phenomenon, we found that in the TMR group, a unique enrichment of many neurotransmitters and their precursors: Dopamine, Noradrenaline, Levodopa, etc., which may point to a new research field: neuro microbiology. In recent years, more and more studies have shown that microorganisms not only have the functions of assisting digestion and maintaining barriers, but also participate in the regulation of the enteric nervous system and the central nervous system by producing neurotransmitters or neurotransmitter precursors (86). It is reported that intestinal microorganisms can synthesize neurotransmitters such as dopamine, norepinephrine, 5-hydroxytryptamine and compounds involved in the metabolism of neurotransmitters in the body, which can act on the local intestinal nervous system and regulate the intestinal environment (87, 88). Taking the synthesis mechanism of dopamine (phenylalanine-tyrosine-L-dopa-dopamine) as an example, phenylalanine hydroxylase converts L-phenylalanine into L-tyrosine, which can cross the blood–brain barrier and enter the brain. In the brain, it is converted to (s)-3,4-dihydroxyphenylalanine (L-DOPA) by tyrosine hydroxylase, and then L-DOPA is converted to dopamine by dopa decarboxylase (89, 90). Although the pathway by which these neurotransmitters are synthesized by the gut microbiome is unclear, it is likely that the gut microbiome also has the ability to convert local dopamine to norepinephrine, such as dopamine-beta oxidase (91). Intestinal microorganisms can also synthesize neurotransmitter precursors such as L-tyrosine. Due to the lack of relevant transporters, some neurotransmitters circulating in the blood, such as dopamine and norepinephrine, cannot cross the blood–brain barrier. However, the precursors of these neurotransmitters can cross the blood–brain barrier, synthesize related substances in the brain, and then regulate, which may be the way that microorganisms connect the central nervous system.

Intestinal flora communicates with the central nervous system, sympathetic nerve and vagus nerve through neurotransmitters, regulates the level of neurotransmitters such as auxin, and affects the growth and development, gastrointestinal digestion and immune function of animals (92). For example, slight changes in the vagus nerve will lead to dramatic changes in the release of downstream neurotransmitters, which will affect digestive level, intestinal permeability, intestinal motility and immune response (93, 94). The central nervous system receives signals from these neurotransmitters and regulates the digestive environment and microbial community composition in the gastrointestinal tract by producing neurochemicals such as 5-hydroxytryptamine, cholecystokinin, and dopamine. Microorganisms also stimulate intestinal endocrine cells to produce hormones and complete feedback to the central nervous system through neural afferent fibers. For example, in the presence of dopamine and norepinephrine, *Escherichia coli* will proliferate faster and may also enhance its motility and toxicity (95). Liu ‘s study found that abnormal casein synthesis aggravated enteritis, disrupted microbial flora and its metabolic function, and led to abnormal immune response (96). The intermediate compound of neurotransmitters: short-chain fatty acids, also participate in the regulation of microorganisms and neurotransmitters. Studies have shown that short-chain fatty acids can regulate the metabolism of many neurotransmitters by acting on the response element binding proteins in the cAMP signaling pathway (97, 98). Taking dopamine as an example, short-chain fatty acids can regulate the production of dopamine and the conversion of dopamine and norepinephrine by regulating the expression of tyrosine carboxylase and dopamine-β-carboxylase (99). These metabolic pathways are reflected in the differential metabolic pathways in the TMR group. Especially for ruminant mammals such as calves, the short-chain fatty acids in the body will be more abundant, and the regulation mechanism of short-chain fatty acids will be more complicated. In some animal model experiments, it has also been found that metabolic abnormalities caused by intestinal microbial community disorders and the release of monoamine substances may lead to depression (100). This proves the deep relationship between intestinal microbial changes and host mental disorders. In animal husbandry, whether it will affect animal feed intake and estrus cycle needs further study.

In recent years, many studies have shown that intestinal microorganisms can communicate and regulate with the gastrointestinal tract and brain through neurotransmitters, and interact to maintain the dynamic balance of the intestinal environment. One of the difficulties in the field of neurobiology is to identify whether the source of various neuroactive compounds is host or microorganism. Secondly, the influence between intestinal microorganisms and neurotransmitters, and the complex communication between intestinal microorganisms and the brain are difficult. Combining the analysis results of metabolomics and metagenomics will help us understand the operation relationship between the microbial-intestinal-brain axis from the perspective of neuro microbiology. In the future, specific microbial targeted interventions can be performed on the host from the perspective of neuro microbiology, which can be used to adjust the intestinal flora and improve digestion. It is even possible to regulate specific microbial communities by understanding the relationship between gut microbes and brain health, and to treat neurological diseases through microbial regulation of the central nervous system.

## 5. Conclusion

In summary, our study found that the feeding mode of ‘starter + hay + milk ‘was more conducive to the development of calves. The calves in the SH group had better intestinal development, intestinal microbial community was more conducive to digestion and absorption, and immunity was stronger. The feeding mode of the SH group was conducive to the healthy development of the calves. Therefore, the calves in the SH group had higher daily weight gain, stronger digestive ability, and no pathological inflammatory reaction was found. For calves, daily weight gain is important, and more importantly, there can be a stronger digestive system and immune system to ensure that calves can have better growth and development after weaning, which is the advantage of SH group compared with SF group. The enrichment of neurotransmitters found in the TMR group also inspired us to study intestinal microorganisms from a new perspective. The research ideas of neuro microbiology will be one of our future focuses. Therefore, the results of this study provide a basis for improving the feeding mode of calves, and inspire us to explore the relationship between microorganisms and hosts from the perspective of neural metabolites.

## Data availability statement

The original contributions presented in the study are included in the article/supplementary material, further inquiries can be directed to the corresponding author.

## Ethics statement

The animal study was approved by the Institutional Animal Care and Use Committee of the College of Animal Science and Technology. The study was conducted in accordance with the local legislation and institutional requirements.

## Author contributions

JW: Data curation, Writing – review & editing, Writing – original draft. YC: Data curation, Writing – original draft, Writing – review & editing. ML: Data curation, Investigation, Project administration, Writing – review & editing. SX: Investigation, Software, Writing – review & editing. KZ: Investigation, Software, Writing – review & editing. HF: Investigation, Software, Writing – review & editing. JN: Investigation, Software, Writing – review & editing. WS: Conceptualization, Investigation, Software, Writing – review & editing. XJ: Conceptualization, Investigation, Software, Writing – review & editing. SL: Methodology, Project administration, Supervision, Writing – review & editing.

## Funding

This study was supported by the key research and development project of Sichuan beef cattle genome selection technology system (2022YFYZ0006).

## Conflict of interest

The authors declare that the research was conducted in the absence of any commercial or financial relationships that could be construed as a potential conflict of interest.

## Publisher’s note

All claims expressed in this article are solely those of the authors and do not necessarily represent those of their affiliated organizations, or those of the publisher, the editors and the reviewers. Any product that may be evaluated in this article, or claim that may be made by its manufacturer, is not guaranteed or endorsed by the publisher.

## Footnotes

^1^ https://github.com/voutcn/megahit

^2^ http://topaz.gatech.edu/GeneMark/

^3^ https://www.ncbi.nlm.nih.gov/

^4^ http://www.kegg.jp/kegg/

^5^ http://www.cazy.org/

^6^ http://www.plob.org/tag/eggnog/
